# Hypercalcemia of Malignancy in Thymic Carcinoma: Evolving Mechanisms of Hypercalcemia and Targeted Therapies

**DOI:** 10.1155/2017/2608392

**Published:** 2017-01-12

**Authors:** Cheng Cheng, Jose Kuzhively, Sanford Baim

**Affiliations:** Division of Endocrinology and Metabolism, Rush University Medical Center, Chicago, IL, USA

## Abstract

Here we describe, to our knowledge, the first case where an evolution of mechanisms responsible for hypercalcemia occurred in undifferentiated thymic carcinoma and discuss specific management strategies for hypercalcemia of malignancy (HCM).* Case Description*. We report a 26-year-old male with newly diagnosed undifferentiated thymic carcinoma associated with HCM. Osteolytic metastasis-related hypercalcemia was presumed to be the etiology of hypercalcemia that responded to intravenous hydration and bisphosphonate therapy. Subsequently, refractory hypercalcemia persisted despite the administration of bisphosphonates and denosumab indicative of refractory hypercalcemia. Elevated 1,25-dihydroxyvitamin D was noted from the second admission with hypercalcemia responding to glucocorticoid administration. A subsequent PTHrP was also elevated, further supporting multiple mechanistic evolution of HCM. The different mechanisms of HCM are summarized with the role of tailoring therapies based on the particular mechanism underlying hypercalcemia discussed.* Conclusion*. Our case illustrates the importance of a comprehensive initial evaluation and reevaluation of all identifiable mechanisms of HCM, especially in the setting of recurrent and refractory hypercalcemia. Knowledge of the known and possible evolution of the underlying mechanisms for HCM is important for application of specific therapies that target those mechanisms. Specific targeting therapies to the underlying mechanisms for HCM could positively affect patient outcomes.

## 1. Clinical Presentation

A 26-year-old African American male, with no significant past medical history, presented to the emergency department in early November 2016 with complaints of fever, malaise, 18 lb weight loss over 2 weeks, and multiple neck masses. Medications prior to admission consisted of cyclobenzaprine, meloxicam, tramadol, and recreational use of marijuana. Initial imaging revealed an anterior mediastinal mass with intrathoracic lymphadenopathy, bilateral pulmonary nodules, and spine lesions on CT.

Physical exam demonstrated bilateral supraclavicular lymphadenopathy that was tender to palpation, pain on palpation of the cervical and lumbar spine, and normal neurological exam.

Labs on admission were notable for corrected total calcium (Calc) of 15.1 mg/dL, ionized calcium (iCa) of 1.59 mg/dL (ref: 0.95–1.32 mg/dL), PTH of 4.8 pg/mL (ref: 8–85 pg/mL), phosphorus (Phos) of 2 mg/dL (ref: 2/5–4.6 mg/dL), creatinine of 1.16 mg/dL (ref: 0.75–1.2 mg/dL), and blood count with no atypical cells seen on the differential. Aggressive IV hydration with normal saline at a rate of 250 cc/hr was promptly started and maintained throughout this admission with administration of pamidronate 90 mg on hospital day 2. Additional studies included supraclavicular lymph node and bone marrow biopsies consistent with Epstein-Barr virus positive metastatic undifferentiated, non-keratinizing, lymphoepithelioma-like carcinoma of thymic origin. After undergoing staging with additional imaging, the patient completed his first cycle of chemotherapy with cisplatin, doxorubicin, and cytoxan in the next 2 weeks. His Calc decreased to 10.5 mg/dL at the time of discharge.

**Figure 1:**
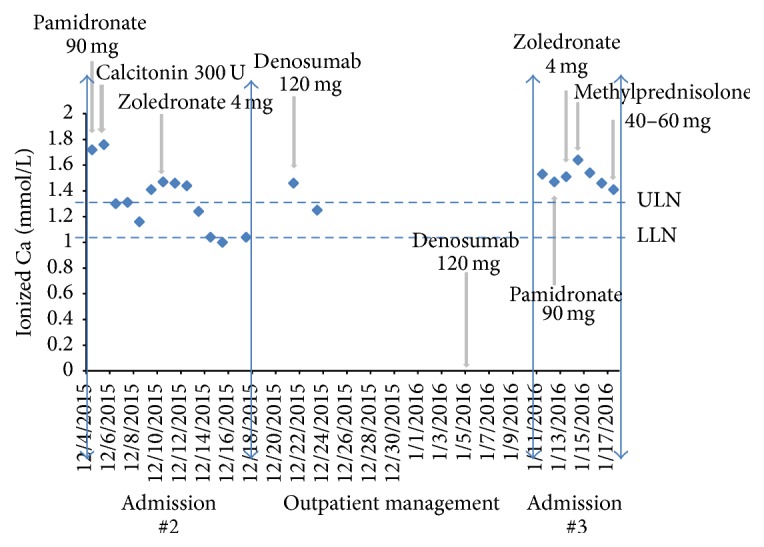
Evolution of hypercalcemia in relation to medical therapies instituted. Please note that majority of ionized calcium data from first admission are unavailable. Also, ionized calcium levels are unavailable on 1/5/2016 when denosumab was administered.

Approximately 2 weeks after discharge, the patient was readmitted for a second admission with increasing somnolence. Laboratory analysis disclosed Calc of 15.4 mg/dL and iCa of 1.72 mg/dL for which IV hydration with normal saline at 250 cc/hr was initiated followed by pamidronate 90 mg and calcitonin 300 U with improvement of iCa to as low as 1.16 mg/dL. PTH-related peptide (PTHrP) and 1,25-dihydroxyvitamin D (calcitriol) were sent during this admission but results were not available. Repeat MRI of the entire spine noted new hyperintense metastatic lesions. Over the ensuing 3 days, iCa slowly increased to 1.46 mg/dL and required administration of zoledronate 4 mg resulting in normalization of iCa between 1 and 1.1 mg/dL for the rest of the admission (Figure 1). The patient subsequently began cycle 2 of cisplatin, doxorubicin, and cytoxan which was completed prior to discharge with a plan to initiate denosumab as an outpatient.

During outpatient follow-up and 5 days after discharge, a rapid rebound in hypercalcemia occurred with Calc of 12.6 mg/dL and iCa of 1.46 mg/dL, requiring administration of denosumab 120 mg which decreased iCa to 1.25 mg/dL (Figure 1). A second dose of denosumab 120 mg was given 1 week later with concurrent Calc of 12.7 mg/dL.

One month later, the patient was readmitted with altered mental status with Calc of 13.6 mg/dL, iCa of 1.53 mg/dL, Phos of 1.6 mg/dL, and normal renal function. The patient received prompt administration of IV hydration with normal saline and pamidronate 90 mg. Although iCa level decreased to 1.3–1.4 mg/dL within 2 days, it rebounded over the next 24–48 hours to 1.64 mg/dL, requiring further administration of zoledronate 4 mg (Figure 1).

At this time, it was noted that his 1,25-dihydroxyvitamin D level from the previous admission was elevated at 131 pg/mL (ref: 18–64 pg/mL) and PTHrP at 27 pg/mL (ref: 14–27 pg/mL). Methylprednisolone 60 mg per day was subsequently instituted over the next 2 days with decrease in iCa level to 1.3–1.4 mg/dL (Figure 1).

However, the patient continued to clinically deteriorate, despite iCa being maintained at 1.3–1.4 mg/dL (Figure 1) with development of multiorgan failure, and he expired shortly after. It is noteworthy that the third admission repeated PTHrP and calcitriol levels that returned to the medical record posthumously were 58 pg/mL and 499 pg/mL, respectively.

## 2. Introduction

Hypercalcemia of malignancy (HCM) commonly presents as the initial manifestation of undiagnosed cancer. HCM is a paraneoplastic syndrome with poor prognosis and up to 50% mortality within the first 2 months of the diagnosis [1, 2]. HCM may be caused by either humoral factors (humoral hypercalcemia of malignancy, HHM) which indirectly enhances bone resorption or direct skeletal invasion by malignant cells (osteolytic metastasis-related hypercalcemia, OMRH). Humoral factors responsible for hypercalcemia are usually PTHrP in 80% of HCM [3] followed by excessive 1,25-dihydroxyvitamin D production by tumor cells or macrophages (calcitriol-induced hypercalcemia, HHM-CIH) in less than 1% [3] and excessive ectopic parathyroid hormone (PTH) producing tumors being rare. Another rare humoral cause is the production of excessive systemic cytokine and/or chemokine induced bone resorption (HHM-SCCBR) with normal PTHrP, calcitriol, and PTH levels and no evidence of OMRH [4]. Usually HCM has a single etiology. Rarely interplay of multiple mechanisms can be the cause [5–8].

The currently elucidated five known mechanisms for HCM and their respective associated cancers are summarized in Table 1. Here we present a case of severe hypercalcemia due to undifferentiated thymic carcinoma involving several hypercalcemia inducing mechanisms that evolved over the course of three admissions. The response of serum calcium to the institution of different therapies based on the identification of the underlying mechanisms is additionally described.

## 3. Discussion

Bisphosphonates, namely, pamidronate and zoledronate, have essentially become the standard therapy following aggressive fluid resuscitation in the management of HCM. The mechanism of action of bisphosphonates in the treatment of HCM is the inhibition of osteoclast-mediated bone resorption, increased osteoclast apoptosis, and decreased osteoblast apoptosis [25, 31]. The rapid rebound of hypercalcemia despite the additional administration of bisphosphonate therapy in our patient, even after his second admission (Figure 1), is consistent with incomplete inhibition of bone resorption [32]. This is often observed with progression of tumor by means of the specific underlying mechanism for HCM whether it be OMRH, PTHrP, or HHM-SCCBR.

The implementation of the novel antiresorptive agent denosumab, a RANKL antibody that inhibits osteoclastic activity, was followed by improvement of iCal to the upper limit of the normal range which persisted until the third admission (Figure 1). This course of action is consistent with findings from recent studies in which the introduction of denosumab is of particular benefit in HCM refractory to bisphosphonates [33]. The recurrent hypercalcemia that prompted our patient's last admission was indicative of both bisphosphonate and denosumab failure but demonstrated dramatic response to glucocorticoid therapy (Figure 1) which is consistent with a different mechanism of HCM or HHM-CIH

The elevated 1,25-dihydroxyvitamin D, as noted in our case, did trigger the prompt administration of prednisone therapy which led to rapid improvement in calcium levels (Figure 1). Although HHM-CIH is widely recognized and studied extensively in granulomatous diseases, increased expression and activity of 1-*α* hydroxylase resulting in overproduction of serum 1,25-dihydroxyvitamin D have also been demonstrated in in vivo studies investigating hypercalcemia associated with dysgerminomas [34] and B-cell lymphoma [35]. The treatment of HHM-CIH is glucocorticoid therapy that inhibits 1-*α* hydroxylase activity, blocking conversion of calcidiol to calcitriol, resulting in decreased absorption of calcium from the intestine, reabsorption of calcium in the renal tubules, and decreased bone resorption [2]. The optimal glucocorticoid treatment dose and duration of therapy remain undefined, with doses ranging from 20 to 400 mg of prednisone or its equivalent administered daily [9, 36].

Hypercalcemia resulting from multiple mechanisms, HHM-CIH and HHM-PTHrP, has been described in rare cases of HTLV-1 positive ATLL [5], neuroendocrine tumors of the pancreas [6], seminoma [7], and ovarian carcinoma [8]. The mechanism elucidated to cause HHM-SCCBR has been described in conjunction with HHM-PTHrP or OMRH, as observed in multiple myeloma and breast cancer [37, 38]. None of these cases illustrated the simultaneous or independent development of multiple mechanisms underlying HCM over time.

Our case is novel in several aspects from other case reports. The first two admissions were presumed to be associated with OMRH, evidenced by extensive bone metastases. The discovery of a progressive elevation of calcitriol over time, refractoriness of treatment with bisphosphonates and denosumab (Figure 1), and significant response to glucocorticoids therapy is consistent with evolution of an alternative mechanism for HCM. The subsequent discovery of a progressive elevation of PTHrP supports an additional mechanism for HCM in this case.

Our case is also unique given the observation of malignancy associated hypercalcemia in undifferentiated thymic carcinoma. To our knowledge, paraneoplastic hypercalcemia has been previously described in only two cases of squamous cell carcinoma of the thymus [10, 11]. The etiology of hypercalcemia, in one of the aforementioned cases, was believed to be secondary to HHM [11].

## 4. Conclusion

Our patient represents the first reported case of the progressive evolution of HCM mechanisms as demonstrated by the findings of refractory and recurrent hypercalcemia associated with discovery of an additional specific mechanism that subsequently responded to the targeted treatment.

In patients presenting with paraneoplastic hypercalcemia, especially in the setting of recurrent or refractory hypercalcemia, it is prudent to evaluate all potential mechanisms of HCM by obtaining measurement of PTH, PTHrP, and calcitriol levels.

## Figures and Tables

**Table 1:** Respective cancers associated with mechanisms of hypercalcemia of malignancy [4, 9–30].

	Hematologic malignancy	Solid organ malignancy
Calcitriol-induced hypercalcemia	(i) Non-Hodgkin's lymphoma (ii) Hodgkin's lymphoma (iii) Chronic lymphocytic leukemia	(i) Gastrointestinal stromal tumor (ii) Glioblastoma multiforme (iii) Metastatic squamous cell carcinoma of tongue (iv) Non-small cell lung carcinoma (v) Metastatic carcinoma of unknown primary (vi) Ovarian dysgerminoma (vii) Renal cell carcinoma (viii) Seminoma

PTHrP-related hypercalcemia	(i) Non-Hodgkin's lymphoma (ii) Chronic myelogenous leukemia (iii) Chronic lymphocytic leukemia (iv) Hodgkin's lymphoma (v) Multiple myeloma (vi) Plasma cell leukemia (vii) Waldenstrom's macroglobulinemia	(i) Squamous cell carcinoma^a^(ii) Adenocarcinoma^b^(iii) Benign congenital mesoblastic nephroma (iv) Bladder cancer (v) Epithelioid hemangioendothelioma (vi) Melanoma (vii) Merkel cell carcinoma (viii) Myxoid sarcoma (ix) Neuroendocrine tumor (x) Seminoma (xi) Uterine leiomyoma

Local osteolysis	(i) Acute lymphocytic leukemia (ii) Multiple myeloma (iii) Non-Hodgkin's lymphoma	(i) Breast cancer (ii) Lung cancer

Ectopic PTH secretion	(i) Acute myelogenous leukemia	(i) Gastric carcinoma
		(ii) Lung cancer
		(a) Small cell
		(b) Squamous cell
		(iii) Neuroendocrine cancer of pancreas
		(iv) Thyroid cancer
		(a) Medullary
		(b) Papillary adenocarcinoma
		(v) Ovarian carcinoma
		(vi) Thymoma
		(vii) Rhabdomyosarcoma

Cytokine-induced hypercalcemia	(i) Acute lymphocytic leukemia	(i) Squamous cell carcinoma of hand
	(ii) Multiple myeloma	
	(iii) Non-Hodgkin's lymphoma	
	(a) Diffuse large B-cell lymphoma	
	(b) Follicular lymphoma	
	(c) Adult T-cell leukemia/lymphoma	

^a^Anus, esophagus, head and neck cancer, lung, manubrium, parotid, penis, skin, scrotum, and vulva [9].

^b^Breast, cholangiocarcinoma, colon, duodenum, endometrium, lung, ovary, pancreas, renal cell, and stomach [9].
